# Quantitative Analysis of Cadmium in Tobacco Roots Using Laser-Induced Breakdown Spectroscopy With Variable Index and Chemometrics

**DOI:** 10.3389/fpls.2018.01316

**Published:** 2018-09-13

**Authors:** Fei Liu, Tingting Shen, Wenwen Kong, Jiyu Peng, Chi Zhang, Kunlin Song, Wei Wang, Chu Zhang, Yong He

**Affiliations:** ^1^College of Biosystems Engineering and Food Science, Zhejiang University, Hangzhou, China; ^2^Key Laboratory of Spectroscopy Sensing, Ministry of Agriculture and Rural Affairs, Hangzhou, China; ^3^School of Information Engineering, Zhejiang A&F University, Hangzhou, China

**Keywords:** cadmium, tobacco root, laser-induced breakdown spectroscopy, interval partial least squares, variable index, multivariate analysis

## Abstract

The study investigated some new developed variable indices and chemometrics for the fast detection of cadmium (Cd) in tobacco root samples by laser-induced breakdown spectroscopy. The variables selection methods of interval partial least squares (iPLS), backward interval partial least squares (BiPLS), and successive projections algorithm (SPA) were used to locate the optimal Cd emission line for univariate analysis and to select the maximal relevant variables for multivariate analysis. iPLS and BiPLS located 10 Cd emission lines to establish univariate analysis models. Univariate analysis model based on Cd I (508.58 nm) performed best with the coefficient of determination of prediction (*R_p_*^2^) of 0.9426 and root mean square error of prediction (RMSEP) of 1.060 mg g^−1^. We developed two new variable indices to remove negative effects for Cd content prediction, including Index1 = (*I*_508.58_ + *I*_361.05_)/2 × *I*_466.23_ and Index2 = *I*_508.58_/*I*_466.23_ based on Cd emission lines at 508.58, 361.05, and 466.23 nm. Univariate model based on Index2 obtained better result (*R_p_*^2^ of 0.9502 and RMSEP of 0.988 mg g^−1^) than univariate analysis based on the best Cd emission line at 508.58 nm. PLS and support vector machines (SVM) were adopted and compared for multivariate analysis. The results of multivariate analysis outperformed univariate analysis and the best quantitative model was achieved by the iPLS-SVM model (*R_c_*^2^ of 0.9820, RMSECV of 0.214 mg g^−1^, *R_p_*^2^ of 0.9759, and RMSEP of 0.712 mg g^−1^) using the maximal relevant variables in the range of 474–526 nm. The results indicated that LIBS coupled with new developed variable index and chemometrics could provide a feasible, effective, and economical approach for fast detecting Cd in tobacco roots.

## Introduction

Cadmium (Cd) is a nonessential and toxic heavy metal for plants, animals, and humans. With the development of modern industry and human activities such as industrial emission and domestic sewage, Cd has been widely spread in agricultural environment and plant-environment system (Dong et al., 2007). The accumulation of Cd may lead to the decrease of yield, affect the quality of plants and endanger human health through the food chain. Root and root hair are almost interspersed in all spaces of soil-plant system and absorb Cd from the polluted soil solution in plant-environment system (Yang et al., 2013). There is no doubt that roots are the usual primary scene exposing to heavy metal in living environments. And some authors pointed out that the greater the heavy mental ionic impulsion in plant roots, the heavier the damage to plant growth (Wang et al., 1994; Khare et al., 2017; Usharani and Vasudevan, 2017). Cd could be easily enriched in tobacco and the proportion of human Cd exposure caused by smoking may exceed the figure from diet, especially for those heavy smokers. Rapid monitoring Cd accumulation in tobacco root is conducive for the detection and supervision of tobacco and land heavy metal pollution timely. However, there is no precedent for fast detection of Cd accumulation on related tobacco research yet.

Laser induced breakdown spectroscopy (LIBS) is an emerging elemental analytical technique based on laser shooting on a sample surface to generate a short pulse of high energy radiation and ablate a little sample to excite a plasma consisting of atomic, ionic, and molecular species (Yi et al., 2017b). Due to the merits of multi-element analysis, fast response, little to no sample treatment and remote sensing, LIBS is now competitive in element detection, compared with other conventional laboratory techniques such as atomic absorption spectrometry (AAS), inductively coupled plasma mass spectrometry (ICP-MS), and inductively coupled plasma optical emission spectrometer (ICP-OES), which are time-consuming and require a very experienced digestion procedure (Jantzi et al., 2016; Yang et al., 2016, 2017). And LIBS also is successful in different applications ranging from space and ocean detection to biological specimens such as the complex plant materials (Santos et al., 2012). However, the application of LIBS in plant materials involving the field of agricultural and environmental sciences is more challenging because of adverse “matrix effects” caused by the complex plant tissue. Matrix effects include changes in chemical composition and physical properties of plant tissue such as hardness, roughness, porosity, density, and moisture (Guezenoc et al., 2017).

The LIBS full spectra of plant samples such as tobacco roots are composed of massive variables containing large amounts of information, including matrix effects information and the experimental conditions fluctuation information such as laser shot-to-shot energy fluctuation and random error of testing samples (Peng et al., 2017). In view of such case, the accuracy and stability of quantification of target element in plant materials are affected and interfered by the vast ineffective variables in the LIBS full spectra. With regard to quantitative analysis, the effective variables are the critical point whether univariate or multivariate procedures are used for LIBS spectral data processing (Fu et al., 2017). The sensitive emission line of target element, which was inquired from National Institute of Standards and Technology Atomic Spectra Database directly performs well in other areas, but may be interfered by other elements and not be suitable in plant materials (Peng et al., 2016). To select the effective variables of target element in specific material tobacco roots, some chemometric methods such as interval partial least squares (iPLS) (Li et al., 2018), backward interval partial least squares (BiPLS) (Zou et al., 2007), and successive projections algorithm (SPA) (Liu et al., 2009) could be attempted. The selected variables could also be combined as new variable index to elevate validity of quantification. The variable selection methods and variable index are adopted mostly in near-infrared spectroscopy (Abrahamsson et al., 2003; Lleo et al., 2011) (molecular spectra) but rarely in LIBS spectra (mainly atomic spectra) analysis. Up to the present, Pontes et al. (2009) considered SPA to select variables for the classification of 149 Brazilian soil samples into three different orders; Fu et al. (2017) proposed a fast variable selection method combining iPLS and modified iterative predictor weighting-PLS for the LIBS quantitative analysis of soils. At the same time, variable selections and variable index in the LIBS spectra of plant materials has not been reported. It is therefore wise to select effective variables of target element Cd and compare different variables combinations to improve LIBS analytical performance for laboratory applications and field supervision of tobacco roots.

In this paper, we studied the feasibility of rapid analysis of Cd accumulation in tobacco root by using LIBS variable index and chemometrics. The specific objectives of this research were (1) to choose suitable variables selection method from iPLS, BiPLS, iPLS-SPA, and BiPLS-SPA, and to obtain outstanding Cd emission lines and the most relevant variables for reference Cd values in tobacco roots; (2) to execute univariate analysis by the selected Cd emission lines and put forward new index based on Cd emission lines to elevate univariate analysis capacity; (3) to compare the prediction performance of PLS and SVM multivariate analysis models based on the selected variables and to find out the optimal variables and the best quantitative models for fast and valid detection of Cd content in tobacco roots.

## Experimental

### Sample Preparation

Hydroponic experiment was carried out on Zijingang Campus, Zhejiang University, Hangzhou, China. Tobacco seeds (MS 87, Yuxi Zhong Yan Seed co., Ltd, China) were used in this study. The sterilized seeds were germinated and cultivated on the Murashige and Skoog culture medium at 30°C for 2 weeks. Then, the seedlings with root length of approximately 4 cm approximately were transplanted into 10 L full strength Hoagland’s nutrient solution and the culture solutions were renewed every 3 days. On the ninth day after transplanting, five treatments were adopted in this experiment with similar size plants, that is, control group and experimental group of 5, 30, 70, and 100 μM Cd stress (prepared by CdCl_2_ solution). The experiment was laid in a completely randomized design with 12 replications for control (CK) and 5 μM Cd stress group, 18 replications for other three treatments (30, 70, and 100 μM) receptively. After 20 days treatment, each tobacco root was collected and soaked in 20 mM Na_2_EDTA solution and then washed by deionized water to clear away free Cd out of root tissue. In total, 80 root samples were collected and dried at 80°C for 4 h in an oven, then were grinded and pressed into pellet separately. Tobacco root powders with 150 mg were placed into a square die set and pressed with 10 tons of pressure for 1 min.

### LIBS Instrumentation

**FIGURE 1:**
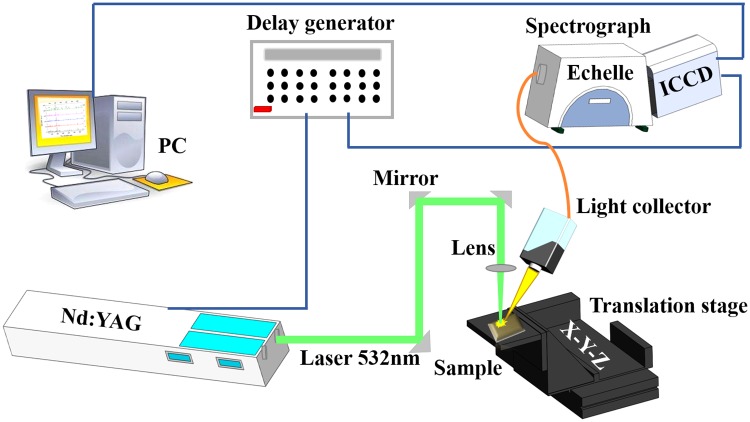
Schematic diagram of the LIBS experimental setup.

A schematic diagram of the self-assembled LIBS setup used in this work is presented in **Figure 1**. Laser pulses were delivered by a Q-switched Nd:YAG pulse laser (Vlite 200, Beamtech, China) at 532 nm with maximum energy of 200 mJ, 8 ns pulse width, repetition rate from 1 to 10 Hz, and 7 mm beam diameter. A plano-convex lens (*f* = 100 mm) was used to focus the excitation laser beam on the samples. The plasma light of ablation was collected by an optical fiber, which delivers light to an Echelle spectrograph coupled with intensified charge coupled device (ICCD) camera (ME5000 and DH334, Andor Technology, United Kingdom) to record signals. The delay time between the ICCD camera and laser Q-switch was controlled by a delay generator (DG645, Stanford Research Systems, United States). The experimental parameters were optimized to obtain the best signal-to-noise ratio at laser energy of 115 mJ, delay time of 4.41 μs, and gate width of 6.48 μs. Individual test pellet was placed on an automatic x–y–z translation stage to supervise the laser ablation position on the sample surface. In this case, the laser beam was focused 2 mm below the sample surface and ablated 4 × 4 array craters. The spectrum for each pellet was recorded by the average of 80 spectra form 16 positions with 5 times accumulation of laser pulses in one position.

### Reference Method for Determining Cadmium Content

Cadmium content in tobacco roots were determined using ICP-OES after microwave digestion (Divya et al., 2017). The pellets after LIBS acquisition were weighed and placed into the TFM vessels with 4 mL of 65% HNO_3_ and 1 mL of 30% H_2_O_2_ for microwave digestion, respectively. After digestion, the cleared digestion solution was translated into 50 mL volumetric flasks and diluted to 30 mL with distilled water by weighing method. Finally, the reference Cd content of solutions was determined with ICP-OES. The Cd values of 78 tobacco root samples were shown in **Table 1**. As shown in **Table 1**, the Cd values in tobacco roots in different Cd-stress levels show statistically significant. Then these reference Cd values were input in regression model with LIBS spectral variables for fast detection of Cd in tobacco roots.

**Table 1:** Reference Cd content of tobacco roots obtained by ICP-OES (mg g^−1^).

Groups	0 μM	5 μM	30 μM	70 μM	100 μM
Number	12	12	18	18	18
Min	0	0.006	1.112	3.128	8.199
Max	0.002	0.030	3.361	9.666	19.048
Mean	0.001	0.015	2.304	6.663	11.577
S.D.	0.006	0.087	0.602	1.735	2.617

*SD, standard deviation.*

### Data Treatment

Wavelet transform (WT) was used to preprocess the raw spectra for reducing the effects of systematic noise. WT decomposes the spectral into low-frequency signals and high-frequency signals (Chen et al., 2014). The principle of WT is to analyze wavelet functions with different spatial and frequency properties. WT with wavelet function Daubechies 6 and decomposition level 3 was used in our study. In addition, by sorting the samples from the lowest to highest according to the reference Cd content values, three in every four samples were selected to a calibration set, and the rest were assigned to prediction set. Thus, 51 and 17 samples were split into the calibration set and prediction set, respectively. The samples in calibration set was employed in the modeling procedures including variables selected by iPLS, BiPLS, SPA for PLS, and SVM calibration models, whereas the prediction set was only used in the final accuracy evaluation of the Cd content prediction models.

**FIGURE 2:**
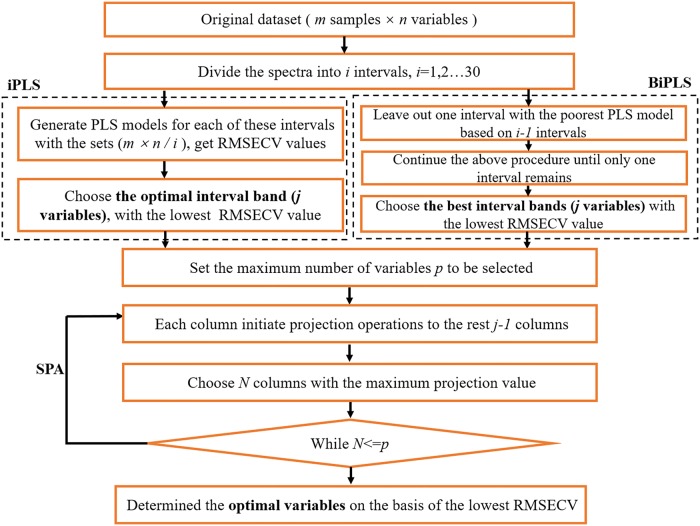
The functions and procedure of the variable fast selection methods iPLS, BiPLS, and SPA.

According to interval variable selection algorithms, iPLS algorithm divides the spectra into several intervals and generates PLS models for each of these intervals (Norgaard et al., 2000). The intervals were formed by continuous emission lines. Only one interval is chosen from the all intervals to establish PLS model for giving the lowest root mean square error of cross-validation (RMSECV) and the highest coefficient of determination (*R*^2^) (Borin and Poppi, 2005). Different from iPLS, BiPLS selects more relevant intervals to explore latent variables (LVs). In BiPLS procedure, PLS models are calculated with each interval left out, that is, if one chooses *k* intervals then compare models based on every different *k*−1 intervals leaving out each interval of *k* intervals and leave out one interval giving the poorest performing model with respect to RMSECV. The rest *k*−1 intervals continue the above procedure until only one interval remains (Wu et al., 2010; Balabin and Smirnov, 2011).The model with the lowest RMESCV based on the best interval variables is investigated. The scheme of variable selection method, which selects the optimal variables in the study is shown in **Figure 2**.

The SPA followed by iPLS and BiPLS was used to select variables with minimum redundant information from the informative intervals selected by the above interval variable selection algorithms (Krepper et al., 2018). The response informative intervals were arranged in a matrix X, with *m* rows (sample number) and *j* columns (LIBS variables accounted *j*−1 column and Cd reference value accounted 1 column) corresponding to the samples and variables, respectively. The main procedures of SPA are summarized (Liu et al., 2009; Milanez et al., 2017) that (1) set the maximum number of variables *p* to be selected, (2) one of *j* columns was yielded to calculate the projection of the remaining *j*−1 column (the process is expressed as projection operations in **Figure 2**), and the columns displaying the least collinearity and maximum projection value were projected onto the orthogonal subspace, (3) if total number of variables in the subspace of the previously selected variable = *p*, restarting (2) procedure from other columns of X, (4) the optimal initial variable and number of variables can be determined on the basis of the smallest RMSECV in a separate validation set.

PLS and SVM were used to provide quantitative analysis and reliable models for explaining the relationship between sample spectral data and true element concentration. As a linear regression method, PLS correlates the maximal variance in independent variables with the dependent variable using regression method (Gottfried et al., 2008; Li et al., 2013). The number of LVs chosen for all the PLS models were optimized by leave-one-out cross-validation in the calibration model. SVM can solve linear and nonlinear regression problems and embody the structural risk minimization principle (Zhang et al., 2015; Yi et al., 2017a). Based on principal components (PCs), the SVM models also applied fivefold cross-validation to get best performances. The radial basis function (RBF) was used as the kernel function of SVM models in this work. All the data analysis was performed in the MATLAB 2014 b (The Mathworks Inc. Natick).

## Results and Discussion

### Raw Spectra Analysis

**FIGURE 3:**
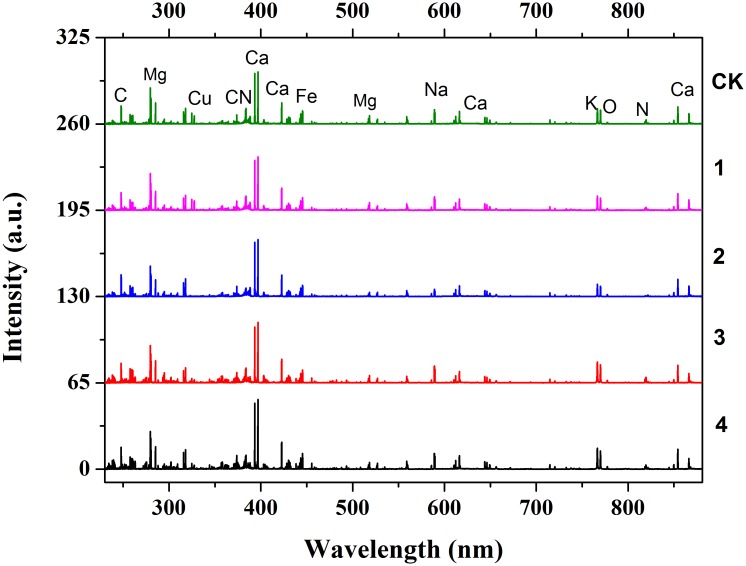
LIBS full spectra after WT of five different Cd stress with additional offset of 65. The five Cd stress from top to bottom is Ck (0 μM), 2 (5 μM), 3 (30 μM), 4 (70 μM), and 5 (100 μM). The obvious emission lines are also marked in CK spectra.

The average raw spectral profiles of five different Cd-stress group samples are shown in **Figure 3**. The patterns of the raw spectra were representative for tobacco roots, with similar basic trends for each group accounting for similar matrix. Based on the Kurucz database and National Institute of Standards and Technology (NIST) Atomic Spectra Database (ASD), some same strong emission lines were observed in all five tobacco root groups such as C (247.86 nm), Mg (279.55, 279.80, 383.82, 516.73 nm), Ca (373.69, 397.37, 82, 422.67, 558.90, 849.80, 866.21, 643.91, 644.98 nm), Cu (324.75, 327.40 nm), CN (393.37, 396.85 nm), Na (589.00, 589.59 nm), and K (766.49, 769.90 nm). These lines included atomic emission lines, ionic emission lines, and molecule bands without spectral interferences and self-absorption phenomenon. The observed emission lines of Fe, Na, Si, Mg, Ca, and K could be used to analyze the variation of micronutrients and macronutrients in tobacco roots. However, some differences were also observed in **Figure 3**. The peak intensity of Ca, Fe, Na, K increased with Cd stress level. Some low-intensity emission lines appeared in the highest Cd-stress group and disappeared in CK group.

### Variable Selection Based on iPLS, BiPLS, and SPA

In this experiment, 22,015 variables per spectrum were acquired in the spectral range of 229.99–880.01 nm with high resolution (λ/Δλ = 5000) and 0.03 nm interval. Too many variables of LIBS spectra with redundancy information may lead to an unpleasant model for quantitative detection of Cd in tobacco roots. The sensitive emission line inquired from NIST ASD may perform poor correlation with Cd content because of complex matrix effect and spectral interference. Meanwhile the urgent demand for fast analysis and online detection need simple variables with less interference and high precision. Therefore, we explored to select effective variables by variable selection methods of iPLS, BiPLS, and SPA.

**FIGURE 4:**
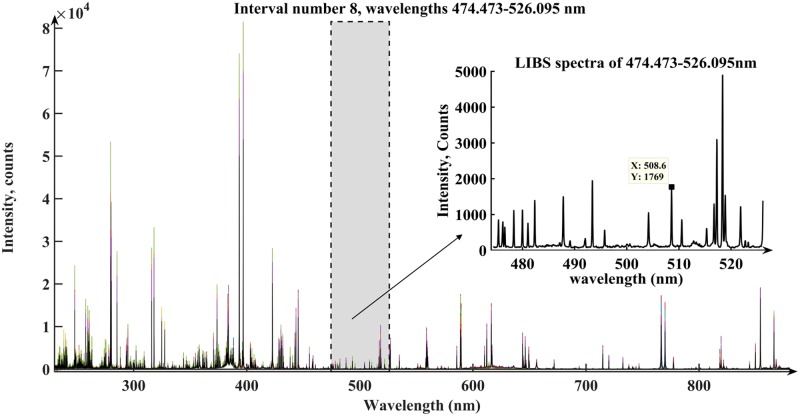
The optimal subinterval and interval selection by iPLS. The selected interval 474.473–526.095 nm obtain the Cd emission lines 508.58 nm (the 1769th variable of the full spectra).

For iPLS, the spectra were divided into *i* (*i* = 2, 3, …, 30) equidistant subintervals and PLS models for each subinterval were established. The best performance of PLS model is based on 1693 variables in the eighth subintervals with *i* = 13 according to the lowest RMSECV value of 0.564 mg g^−1^ as shown in **Supplementary Table S1**. The optimal subinterval was corresponding to interval 474–526 nm (**Figure 4**). Compared with the full spectra, the number of variables in the subintervals *i* = 13 made a big difference. The RMSECVs reduced from 1.360 to 0.564 mg g^−1^, while the number of variables was reduced from 22,015 to 1693. It turned out that in most case the full LIBS spectra have plenty of invalid data for Cd content and iPLS provides an overview of the relevant information of different spectral subdivisions to excavate hidden variables.

As for BiPLS, the full spectra were divided into *i* (*i* = 2, 3, …, 30) equidistant subintervals the same as the iPLS method. The first model was built based on the rest intervals after leaving out one interval. The second model was based on the rest intervals after leaving out the second interval. This procedure continued until only one interval left. Each total subintervals (*i*) obtained its lowest RMSECV after all cycle processes. When *i* = 28, BiPLS model achieved the lowest RMSECV value of all subintervals numbers (*i*). The detailed results for *i* = 28 are also shown in **Table 2**. The first discarded interval for *i* = 28 was the number 1 because of the poorest performing PLS model based on the rest intervals. The selected intervals were intervals [22, 16, 17, 19, 15, 9, 8, 11, 10] with respect to the optimized RMSECV value 0.690 mg g^−1^. The optimal subintervals were corresponding to intervals 323–390 nm, 451–521 nm, 523–546 nm, and 631–662nm. The variable number decreased from 22,015 to 7074 after variable selection based on BiPLS. BiPLS eliminated the corresponding noise intervals and extracts effective variables to establish the base model at every step (Ren et al., 2016).

**Table 2:** Selection of the most efficient interval regions by BiPLS for reference Cd values in tobacco roots.

BiPLS *i* = 28				BiPLS *i* = 28			
Interval Number	Removed Interval	RMSECV	Numbers	Interval Number	Removed interval	RMSECV	Numbers
28	1	1.163	22015	14	25	0.739	11004
27	4	1.121	21228	13	21	0.712	10218
26	28	1.081	20441	12	18	0.699	9432
25	20	1.055	19655	11	23	0.692	8646
24	5	1.027	18869	10	24	0.691	7860
23	3	0.987	18082	9	22	0.690	7074
22	12	0.966	17295	8	16	0.691	6288
21	27	0.932	16509	7	17	0.693	5502
20	7	0.905	15723	6	19	0.700	4716
19	26	0.885	14936	5	15	0.700	3930
18	2	0.867	14150	4	9	0.710	3144
17	6	0.824	13363	3	8	0.744	2358
16	13	0.777	12576	2	11	0.971	1572
15	14	0.757	11790	1	10	0.870	786

**FIGURE 5:**
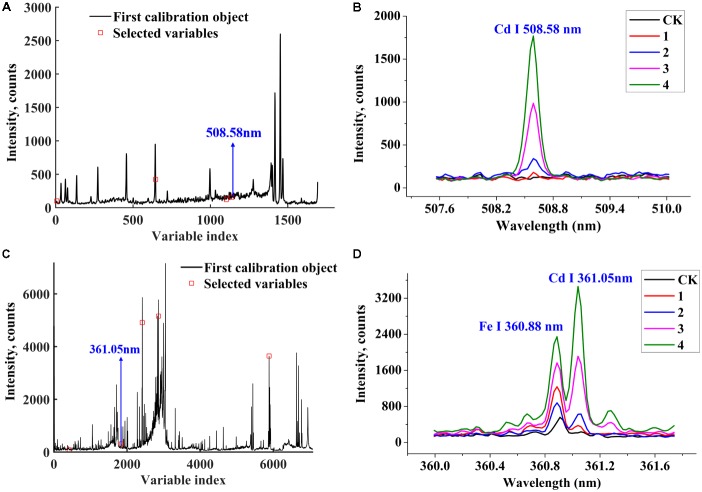
The four optimal variables (474.64, 493.51, 507.47, and 508.58 nm) selected by iPLS-SPA **(A)** and the selected Cd I (508.58 nm) emission lines among the varieties; **(B)** the five optimal variables (331.24, 361.05, 373.69, 383.82, and 558.90 nm) selected by BiPLS-SPA **(C)** and the selected Cd I (361.05 nm) emission lines among the varieties **(D)**.

The LIBS spectra selected by iPLS or BiPLS may have a high level of collinearity and useless information. It will have a negative effect on the prediction performance of the Cd content calibration model. In contrast, SPA have been successfully employed to minimize collinearity problems (Pontes et al., 2009). At the first step of SPA, permitted maximum number was set to 30. **Figures 5A,C** shows the robust variables selected by SPA from spectra intervals after iPLS and BiPLS procedures, respectively.

iPLS-SPA selected four variables (474.64, 493.51, 507.47, and 508.58 nm) for Cd content analysis, where the RMSECV reached its lowest value (0.657 mg g^−1^). According to NIST, it could be obviously seen that 508.58 nm is the atomic emission line of Cd and the characteristic peak of Cd in **Figure 5B** is pure and not influenced by other lines. Obviously, the intensity of Cd I (508.58 nm) lines were increased with the Cd-stress aggravated and the Group 4 had the highest emission intensity of Cd without self-absorption. The intensity of 474.64 and 507.47 nm revealed that the two variables were close to stable background signals, as shown in **Figure 5A**.

In **Figure 5C**, BiPLS-SPA selected five variables (361.05, 373.69, 558.90, 383.82, and 331.24 nm) with the lowest RMSECV value of 0.543 mg g^−1^. The characteristic peak at 361.05 nm is the frequently used sensitive line Cd I. Clearly, Fe I (360.88 nm) is close to the LIBS excited line Cd I (361.05 nm), and there is a small gap between Fe line and excited Cd line, as shown in **Figure 5D**. The iron atoms may disturb ablation energy absorption of the Cd atoms. The intensity differences of Cd I (361.05 nm) lines and four other variables are shown in **Figure 5C**. Signal at 331.24 nm had the lowest intensity and belonged to smooth and steady background signal. Ca and Mg are macronutrients of tobacco roots. Peaks at 373.69, 558.90, and 383.82 nm belonged to Ca I, Ca I, and Mg I according to NIST and had strong intensities. Peaks at 373.69, 558.90, and 383.82 nm were far from Cd I (361.05 nm) and had no interference. The spectral lines at 373.69 nm (Ca I), 558.90 nm (Ca I), 383.82 nm (Mg I), and background signal at 331.24 nm chosen by BiPLS-SPA may improve the analytical sensitivity of Cd I (361.05 nm).

### Univariate Analysis Based on Cd Emission Lines and Variable Index

Univariate analysis is a traditional calibration method and generates the calibration curve by relating the reference element content values with spectral intensities. The ideal univariate analysis is that the intensities of emission lines are proportional to the interested element (Cd) content with no shot-to-shot fluctuation, interruption of other emissions, and matrix effect. As mentioned earlier, the iPLS selected interval 474–526 nm and BiPLS selected intervals 323–390 nm, 451–521 nm, 523–546 nm, and 631–662 nm. According to the above-selected intervals, our study recognized 10 atomic emission lines of Cd I (326.10, 340.36, 346.61, 361.05, 361.28, 361.44, 466.2, 467.81, 508.58, and 643.84 nm) by consulting NIST and referring to the relative intensities of ions. The univariable calibration and prediction results of the above Cd lines were shown in **Table 3**.

**Table 3:** The results for univariate analysis with different Cd atomic emission lines.

Cd Lines (nm)	Calibration Set		Prediction Set	
	*R_c_*^2^	RMSECV mg g^−1^	*R_p_*^2^	RMSEP mg g^−1^
326.10	0.9578	0.979	0.9160	1.391
340.36	0.9366	1.204	09089	1.372
346.61	0.9517	1.051	0.9152	1.282
361.03	0.9600	0.952	0.9185	1.269
361.28	0.9350	1.222	0.8609	1.720
361.44	0.6286	2.912	0.4968	3.159
466.23	0.0544	4.657	0.1867	3.993
467.81	0.8951	1.545	0.8926	1.566
508.58	0.9684	0.846	0.9426	1.060
643.84	0.4902	3.447	0.5181	3.084

The Cd intensity data at 361.05 nm also showed an obvious correlation with reference Cd content in tobacco roots. While the Cd quantitative model based on 508.58 nm had the highest correlation coefficients and lowest RMSE for calibration and prediction set. One reason is that the Cd I (361.05 nm) line was adjacent to Fe I (360.88 nm) having strong and stable intensity in the **Figure 5D**. Fe is the micronutrients of plant and belongs to the matrix atoms of tobacco roots. Fe atoms absorbed more laser energy so that the laser energy absorbed by the atoms of Cd lessened and the analytical sensitivity of target element Cd worsened (Li et al., 2017). The Cd lines in 508.58 nm were pure and nondisruptive. The results were corresponding to the variables selected by iPLS-SPA (508.58, 493.51, 474.64, and 507.47 nm) and BiPLS-SPA (361.05, 373.69, 558.90, 383.82, and 331.24 nm) respectively.

In molecular spectra analysis, spectral indices were proposed to explore the range of pigments such as chlorophyll and anthocyanin in normal conditions (Peng and Lu, 2007; Qin and Lu, 2008). Lleó et al. (2011) pointed that the indices may consist of a single reflectance wavelength, differences or ratios between wavelengths or derivatives. We attempted to propose new indices based on the selected Cd emission lines. And no significant correlation is observed between intensity of Cd I (466.23 nm) and Cd reference content, as shown in **Table 3**. It means that the signal of 466.23 nm presented a narrowing effect and was severely influenced by background noise, matrix effect, sensors, or other circumstances. Our paper proposed two new indices of Cd content prediction based on 508.58, 361.05, and 466.23 nm:

(1) $$\mathrm{Index}\;1=(I_{508.58}+I_{361.05})/(2*I_{466.23})$$

(2) $$\mathrm{Index}\;2=I_{508.58}/I_{466.23}$$

**FIGURE 6:**
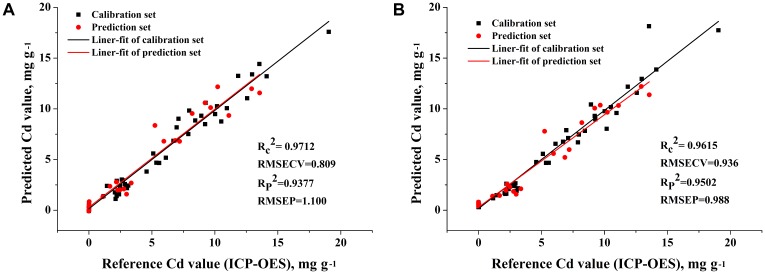
The relationship between reference Cd value and LIBS measured Cd value that predicted by **(A)** Index1 analysis and **(B)** by Index2 analysis.

The two indices were applied for linear regression with reference Cd content values and the regression coefficients were shown in **Figure 6**. The calibration model of Index1 with *R_c_*^2^ value of 0.9712 and RMSECV value of 0.809 mg g^−1^ performed better than any other calibration model based on Cd emission line 508.58 nm or 361.05 nm. The prediction model of Index2 with *R_p_*^2^ value of 0.9502 and RMSEP value of 0.988 mg g^−1^ behaved better than the best univariate analysis based on Cd emission line at 508.58 nm. The results revealed the indices based on LIBS spectral variables were appropriate for univariate analysis of Cd content in tobacco roots.

### Multivariable Analysis Based on Chemometrics

Multivariate analysis is capable to combine useful multi-variables to deal with matrix effect and shot-to-shot fluctuation of LIBS spectral. In recent years, the chemometric methods such as partial least squares (PLS) and support vector machines (SVM) have been extensively used in LIBS spectra for multivariable analysis (Zhang et al., 2014; Yan et al., 2017).

The full spectra and the spectral variables selected by iPLS, BiPLS, iPLS-SPA, and BiPLS-SPA were input into the PLS and SVM models, respectively. The efficiency of PLS and SVM models were all evaluated according to RMSECV, the lowest root mean square error of prediction (RMSEP), and the highest correlation coefficient square (*R*^2^) of calibration set and prediction set (*R_c_*^2^ and *R_p_*^2^).

**FIGURE 7:**
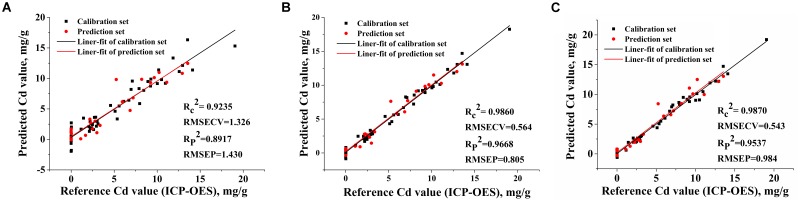
The relationship between reference Cd value and LIBS measured Cd value that predicted by PLS models based on **(A)** 22015 variables of the full spectra; **(B)** 1693 variables selected by the iPLS; **(C)** five variables selected by the BiPLS-SPA.

The performance of the PLS calibration models and prediction models for Cd content in tobacco roots were obtained and summarized in **Table 4**. Among the four variable selection patterns, the full spectra PLS model was the worst with the lowest *R_c_*^2^ and *R_p_*^2^ value and highest RMSECV and RMSEP, and this result indicated that the full spectra data contained massive redundant information leading to bad prediction results; The PLS models based on the four variables selected by iPLS-SPA and the five variables selected by BiPLS-SPA performed well with similar results of *R_c_*^2^ > 0.98 and *R_p_*^2^ > 0.95; The 1693 variables selected by iPLS showed the best quantitative result and indicated that variables selected by BiPLS still reserved invalid information and iPLS-SPA and BiPLS-SPA removed the effective variables of Cd values in tobacco roots. **Figure 7** shows the calibration and prediction plots of the full spectral PLS model and the top two PLS model based on iPLS and BiPLS-SPA selected variables. The BiPLS-SPA model was found to fit reasonably well with *R_c_*^2^ value of 0.9870 and *R_P_*^2^ value of 0.9537 for 5 LVs. The iPLS model showed best linearity, with *R_c_*^2^ value of 0.9860 and *R_p_*^2^ value of 0.9668 for 11 LVs.

**Table 4:** The results for multivariate analysis by PLS and SVM with different variable selection methods.

Variable Selection Methods	Model	Number	Factor	Calibration Set		Prediction Set	
				*R_c_*^2^	RMSECV mg g^−1^	*R_p_*^2^	RMSECP mg g^−1^
	PLS	22015	8	0.9235	1.326	0.8917	1.430
	SVM	22015	13	0.9294	1.271	0.9005	1.372
iPLS	PLS	1693	11	0.9860	0.564	0.9668	0.805
	SVM	1693	13	0.9820	0.214	0.9759	0.712
BiPLS	PLS	7074	10	0.9795	0.691	0.9262	1.35
	SVM	7074	13	0.9994	0.110	0.9743	0.713
iPLS-SPA	PLS	4	4	0.9810	0.657	0.9512	0.997
	SVM	4	4	0.9880	0.521	0.9539	1.04
BiPLS-SPA	PLS	5	5	0.9870	0.543	0.9537	0.984
	SVM	5	5	0.9946	0.349	0.9666	0.891

*Factor for PLS was latent variable, and factor for SVM was PCs.*

**FIGURE 8:**
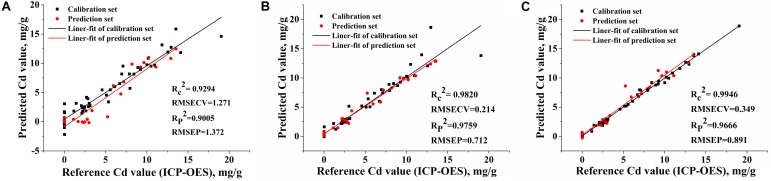
The relationship between reference Cd value and LIBS measured Cd value that predicted by SVM models based on **(A)** 22015 variables of the full spectra; **(B)** 1693 variables selected by the iPLS; **(C)** five variables selected by the BiPLS-SPA.

As mentioned previously, **Table 4** and **Figure 8A** shows that the full spectra data owing interference information lead to a bad SVM modeling effect. After variable selection, new and reduced spectral matrix was generated by selecting the LIBS spectra only at the most important variables that contained the most relevant spectral information of Cd content in tobacco roots.

As shown in **Table 4**, the SVM model based on the 1693 variables selected by iPLS obtained the best quantitative result with *R_c_*^2^ value of 0.9820 and *R_p_*^2^ value of 0.9759 for 13 PCs, and the prediction result has been displayed in **Figure 8B**. The SVM models using the four and five variables selected by iPLS-SPA and BiPLS-SPA (**Figure 8C**) were more simplified models with high accuracy and benefit to develop portable instrument for Cd content fast detection in tobacco field and workshop. In addition, the results of SVM models outperformed those of PLS models, as shown in **Table 4**. It was mainly attributed to the ability of SVM to deal with nonlinear information caused by the “matrix effect” and complex ablation processing. The results demonstrated that new matrices formed by variables selection methods (especially iPLS) could replace the full range spectra to build SVM models to determine Cd in tobacco roots.

Multivariate analysis except using full spectra as input was superior to univariate analysis in terms of calibration and prediction correlation. Multivariate analysis had the capability to deal with specialized features that might be caused by laser-to-sample interaction, the variance of experimental parameters, matrix effect, and so on. Taking spectral selectivity and sensitivity into account, multivariate analysis was more suitable for accurate detection of Cd content in tobacco roots for meticulous laboratory research. On the other hand, univariate analysis based on the new indices and multivariate analysis based on variables selected SPA-iPLS and SPA-BiPLS were more beneficial for exploiting portable instrument for rough fieldwork. At the stage of accurate detection, our method shows the ability of rapid detection for Cd content in tobacco roots. The whole sample pretreatment for acquisition of LIBS signals was less than 5 min including grinding and pressing, while the pretreatment for ICP-OES procedure needs more than 150 min and contains weighting, adding other reagent, digesting, discharging acid, diluting, and so on. After sample pretreatment, time of LIBS information collection for one sample is about 1 min and is compatible with the requirements of on-site analysis. The accuracy and rapidness of LIBS technique combining with variable index and chemometrics provide an accurate assessment for heavy metal Cd content of tobacco roots in a short period of time. The method also benefits for quickly analyzing pollution levels of soil contacting the roots and real-time monitoring the growth of tobacco plants.

## Conclusion

In this experiment, we have shown the potential of LIBS to rapidly detect heavy metal Cd in tobacco root samples with good accuracy results. Our study located the optimal variables by the feature selection methods iPLS, BiPLS, and combinations of the two methods with SPA (iPLS-SPA and BiPLS-SPA). The variables selected by four variables selection methods all obtained low RMSECV and good correlations of reference Cd content. iPLS-SPA and BiPLS-SPA selected the nonoverlapped atomic emission line Cd I (508.58 nm) and the high intensity line Cd I (361.05 nm), respectively. Univariate analysis models were established by the ten Cd emission lines within the variables selected by iPLS and BiPLS. Among the 10 Cd lines, Cd I 508.58 nm performed best with the *R_p_*^2^ of 0.9426 and RMSEP of 1.060 mg g^−1^. Two indices based on 508.58, 361.05, and 466.23 nm were proposed to improve the univariate analysis ability for Cd content prediction and remove some negative impact form noses, then Index2 obtained the better result with the predicted correlation coefficient of 0.9502 and RMSEP of 0.988 mg g^−1^. In addition, PLS and SVM were adopted for multivariate analysis based on full spectra and selected variables. SVM models outperformed PLS models. The best prediction result was achieved by the iPLS-SVM model (*R_c_*^2^ = 0.9820, RMSECV = 0.214 mg g^−1^, *R_p_*^2^ = 0.9759, RMSEP = 0.712 mg g^−1^) with the variables in the range of 474–526 nm.

The proposed approach provided a fast locating method for effective variables. Then the heavy metals in biological samples were quantified by the effective LIBS variables based on the appropriate multivariate analysis models precisely. The proposed approach is simple and efficient, and it is available for element detection in biological samples such as roots and tubers food. Besides, the proposed indices were available for the development of portable instrument detecting Cd contamination in harsh field. Nevertheless, further advances on the basis of our study are still needed. The accumulation of the same heavy metals in different parts of plants such as leaf and stem can be explored for matrix differences and more samples with other chemometric methods can be attempted to develop more robust and precise models and indices.

## Author Contributions

FL, TS, KS, and YH conceived and designed the experiments. FL, TS, KS, JP, and WW performed the experiments. FL, TS, WK, CiZ, and CuZ analyzed the data. FL and YH contributed reagents, materials, and analysis tools. FL, TS, and YH wrote the paper.

## Conflict of Interest Statement

The authors declare that the research was conducted in the absence of any commercial or financial relationships that could be construed as a potential conflict of interest. The reviewer DD and the handling editor declared their shared affiliation.

## Supplementary Material

TABLE S1 Selection of the first 11 most efficient interval by iPLS.
