# Supplementary material for: Quantitative Analysis of Cadmium in Tobacco Roots Using Laser-Induced Breakdown Spectroscopy With Variable Index and Chemometrics
Source: Front Plant Sci. 2018 Sep 13;9:1316. doi: 10.3389/fpls.2018.01316 (PMC6146896; doi:10.3389/fpls.2018.01316)
Supplement: TABLE S1 — Selection of the first 11 most efficient interval by iPLS. [file Table_1.DOCX]

Supplementary Material

Quantitative analysis of Cadmium in tobacco roots using laser-induced breakdown spectroscopy with variable index and chemometrics

Fei Liu^1,3^, Tingting Shen^1^, Wenwen Kong^1,2^, Jiyu Peng^1^, Chi Zhang^1^, Wei Wang^1^, Chu Zhang^1^, Yong He^1,3*^

*** Correspondence: Yong He**: yhe@zju.edu.cn

## Supplementary Table

**Table S-1** Selection of the first 11 most efficient interval by iPLS.

| ***i*** | **selected interval** | **number** | **RMSECV**  **mg g^-1^** | ***i*** | **selected interval** | **number** | **RMSECV**  **mg g^-1^** |
| --- | --- | --- | --- | --- | --- | --- | --- |
| 1 | 230-880 nm | 22015 | 1.360 | 17 | 507-548 nm | 1295 | 0.592 |
| 10 | 450-515 nm | 2202 | 0.605 | 18 | 485-523 nm | 1224 | 0.627 |
| 11 | 478-541 nm | 2002 | 0.586 | 20 | 481-515 nm | 1101 | 0.596 |
| 12 | 503-563 nm | 1835 | 0.602 | 22 | 478-509 nm | 1001 | 0.644 |
| 13 | 474-526 nm | 1693 | 0.564 | 26 | 499-526 nm | 847 | 0.632 |
| 15 | 471-515 nm | 1468 | 0.614 | 29 | 505-529 nm | 760 | 0.636 |
